# Sintilimab maintenance therapy post first-line cytokine-induced killer cells plus chemotherapy for extensive-stage small cell lung cancer

**DOI:** 10.3389/fonc.2022.852885

**Published:** 2022-09-09

**Authors:** Baozhen Ma, Yu Zhou, Yiman Shang, Yong Zhang, Benling Xu, Xiaomin Fu, Jindong Guo, Yonghao Yang, Fang Zhang, Mengyuan Zhou, Hao Huang, Fanghui Li, Hongwei Lin, Lingdi Zhao, Zibing Wang, Quanli Gao

**Affiliations:** Department of Immunotherapy, Affiliated Cancer Hospital of Zhengzhou University, Zhengzhou, China

**Keywords:** extensive-stage small-cell lung cancer, cytokine-induced killer cells, chemotherapy, sintilimab, maintenance

## Abstract

Despite recent progress in treating advanced non-small cell lung cancer, clinical intervention in extensive-stage small-cell lung cancer (ES-SCLC) remains stagnant. The purpose of this study was to evaluate the clinical efficacy of cytokine-induced killer (CIK) cells combined with cytotoxic chemotherapy, followed by anti-programmed death 1 antibody (sintilimab) maintenance, in ES-SCLC patients. To explore a new method for safe treatment of ES-SCLC patients, thirteen ES-SCLC patients were enrolled between June 2019 and December 2021. All patients received first-line chemotherapy (etoposide plus platinum) combined with CIK cell therapy. Patients who reached a stable disease state or responded well to treatment received sintilimab maintenance treatment. The primary objective of this study was to determine the median overall survival (OS); the secondary objective was to assess the objective response rate (ORR), progression-free survival 1 and 2 (PFS1 was defined as the duration from the signing of informed consent to the date of tumor progression, or death, or the last follow-up. PFS2 was defined as the duration from the first day of sintilimab treatment to the date of tumor progression, death, or the last follow-up.), and adverse reactions. At a 24.1-month follow-up, the median OS was 11.8 (95% confidence interval [CI]: 10.6–13.0) months, median PFS1 was 5.5 (95% CI: 5.0–6.0) months, and the median PFS2 was 2.3 (95% CI: 0.5–4.1) months. The ORR was 76.9% (10/13), the disease control rate was 100% (13/13), and the 20-month survival rate was 41.7%. Eight participants exhibited grade 3 or 4 adverse events after combination therapy. During maintenance treatment with sintilimab, level 3 adverse events occurred in 1 patient (1/9). In conclusion, adding CIK cells to standard chemotherapy regimens, followed by maintenance therapy with sintilimab, may represent a new safe and effective treatment strategy.

**Clinical trial registration:**

ClinicalTrials.gov (NCT03983759)

## Introduction

Lung cancer is characterized by malignant tumors and has the highest morbidity and mortality rates among tumorous cancers, with small cell lung cancer (SCLC) being the most aggressive subtype. Furthermore, approximately two-thirds of SCLC patients are diagnosed in advanced stages of the disease (1). Standard first-line treatment for extensive-stage small-cell lung cancer (ES-SCLC) includes platinum (cisplatin or carboplatin) combined with etoposide (EP/EC) chemotherapy, which has remained unchanged for more than three decades until the advent of immunotherapy (2, 3). The objective response rate (ORR) of EP/EC in ES-SCLC can be as high as 70%; however, almost all cases inevitably develop resistance to chemotherapy. The median overall survival (OS) of patients ranges from 7–10 months, with 2- and 5-year survival rates of < 5% and < 2%, respectively (4–8).

The emergence of immune checkpoint inhibitors (ICIs) has changed the treatment approach for many malignancies. With the Food and Drug Administration (FDA) approval of carboplatin, etoposide, and the anti-programmed death-ligand-1 (PD-L1) antibodies atezolizumab or durvalumab, as first-line therapy options, and the anti-programmed cell death protein-1 (PD-1) antibodies pembrolizumab and nivolumab as monotherapies in third-line settings, ES-SCLC treatment has entered the era of immunotherapy (9, 10). Specifically, with the addition of the anti-PD-L1 antibody into the first-line therapy setting, the median OS of patients with ES-SCLC has increased to more than one year for the first time. However, the median OS for patients with ES-SCLC is still less than 13 months, and therapeutic outcome for patients remains dismal. Therefore, exploring alternative therapeutic regimens for ES-SCLC patients is necessary.

Cytokine‐induced killer (CIK) cells, a type of adaptive immunotherapy, involves a group of heterogeneous cell types, represented by CD3^+^CD56^+^ and CD3^+^CD8^+^ subsets (11, 12). Preliminary clinical studies have shown that CIK cells have a wide range of anti-tumor effects, minimal adverse events (AEs), and synergistic anti-tumor properties when combined with traditional treatments, including chemotherapy and immune checkpoint inhibitors (ICIs) (11, 13–19). Ding et al. (20) reported that the use of cellular immunotherapy, including CIK cells, as maintenance therapy for SCLC can prolong the survival of patients. Huang et al. (21) reported positive results in the treatment of ES-SCLC with chemotherapy plus CIK cells. Generally, most patients with ES-SCLC are sensitive to chemotherapy with reduced tumor burden, which reshapes the immunosuppressive microenvironment created by the tumor, and confers the response to ICIs (22–24). In addition, chemotherapy can prevent tumor-induced immune suppression by activating immune effectors, such as natural killer cells (25). Theoretically, CIK cells, combined with other clinical agents, could significantly improve the prognosis of ES-SCLC patients. However, the existing studies on the first-line treatment of ES-SCLC mainly evaluated different combinations of chemotherapy agents, ICIs and tyrosine kinase inhibitors (TKIs). Other immunotherapeutic strategies are being recently reported, including chimeric antigen receptor (CAR) T-cell therapy, tumor vaccines, antibody-drug conjugates (ADCs) and immunomodulators. CIK cells are rarely included in the protocol. To investigate this, we performed phase II clinical studies in treatment-naive ES-SCLC patients to explore the efficacy of chemotherapy plus CIK cells, followed by sintilimab maintenance therapy.

## Materials and methods

### Patients and the database

This study is a single-arm, single-center, open, prospective Phase II clinical study conducted at the Affiliated Cancer Hospital of Zhengzhou University, Henan, China. According to the guidelines of the Declaration of Helsinki, the Ethics Committee of the Affiliated Cancer Hospital of Zhengzhou University reviewed and approved the study. Written informed consent was obtained from all participants. This study was registered at ClinicalTrials.gov (NCT03983759).

Inclusion criteria included: (1) age ≥18 years, ≤70 years, without previous systemic treatment; (2) diagnosed by histology or cytology combined with imaging (the Veterans Administration Lung Study Group staging system); (3) expected survival time >3 months; (4) Eastern Cooperative Oncology Group performance-status score of 0–1; (5) at least one measurable lesion according to the Response Evaluation Criteria in Solid Tumors version 1.1(RECIST 1.1); (6) sufficient organ function; and (7) no other serious diseases (such as autoimmune diseases, immunodeficiency, organ transplantation) that conflict with the study plan. The exclusion criteria were as follows: (1) severe infectious diseases within 4 weeks of enrollment; (2) use of immunosuppressive agents, specifically ≥10 mg/day oral prednisone for more than 2 weeks, before enrollment due to coexisting conditions; and (3) pregnancy or breastfeeding.

### CIK cell preparation and treatment regimens

The procedure for CIK cell preparation is as follows (18); First, 50 mL of heparinized peripheral blood was collected from each patient. Peripheral blood mononuclear cells (PBMCs) were isolated by centrifugation and inoculated into flasks previously treated with RetroNectin (Takara, Japan) and CD3 antibody (Takara). Second, PBMCs were cultured with GT-T551 serum-free medium (Takara) supplemented with 2% autologous plasma, 1000 U/mL IL-2, and 1000 U/mL IFN​​-γ. After culturing for 4 days, the cells were collected and transferred to GT-T610 culture bags (Takara), and a fresh medium containing 1000 U/mL IL-2 was added to the culture bags every 3 days. CIK cells were harvested between day 12 and day 14. CIK cells viability was tested using trypan blue staining. Bacteria and fungi contamination was examined using Becton Dickinson BACTEC 9120 Blood Culture System. Mycoplasma contamination was tested using PCR assay (Bio-Rad, My-Cycler™). Endotoxin contamination was tested using Endotoxin Test Kit (Xiamen Bioendo Technology Co., Ltd).

**Figure 1:**
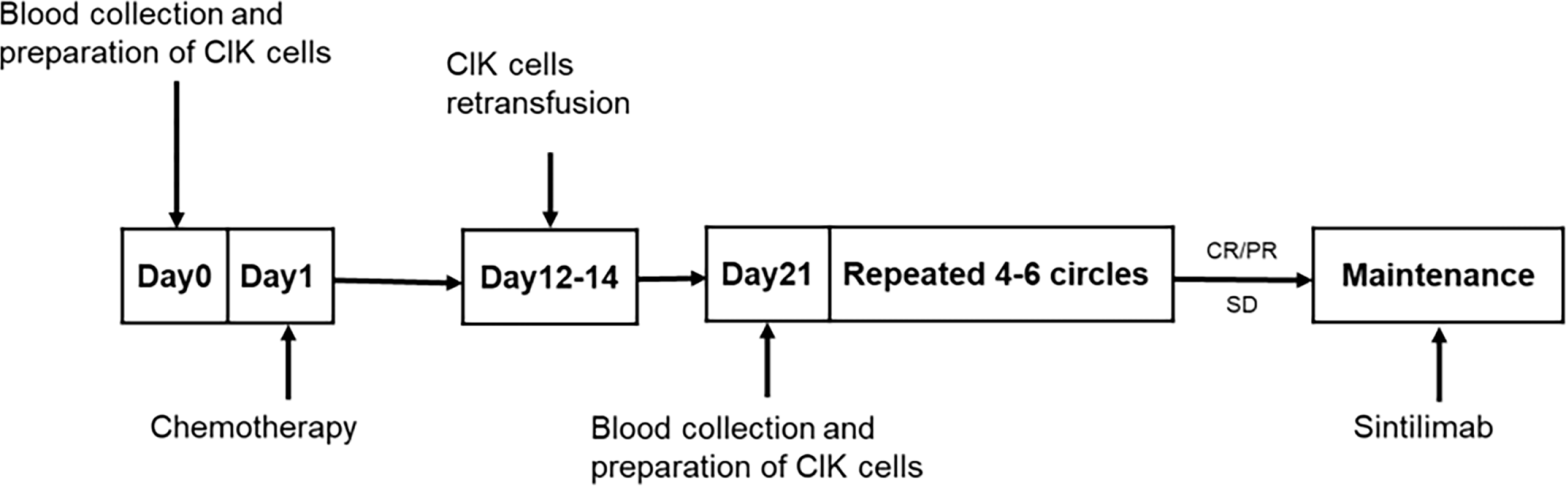
Flow chart of ES-SCLC patient treatment.

Next, 50 mL of peripheral blood was drawn 1–7 days before each chemotherapy treatment for CIK cells culture. All drugs were administered intravenously. EP/EC consisted of etoposide 100 mg/m² on days 1–3 of each cycle with the investigator’s choice of either cisplatin, administered at 75 mg/m², or carboplatin, area under the curve at 5 mg/mL per min, on day 1 of each cycle. CIK cells were infused on days 2–7 after the end of each cycle of chemotherapy. The number of CIK cells per infusion was approximately 5×10^9^. A cycle consisted of 21 days, and a full review and an evaluation were completed after every two cycles. After 4–6 cycles of treatment, patients with complete response (CR), partial response (PR), or stable disease were evaluated and provided maintenance therapy using sintilimab. If a participant exhibited disease progression or intolerable side effects during combined chemotherapy and immune cell therapy, treatment was switched to a second-line approach including irinotecan plus CIK cells after 2–4 cycles, followed by sintilimab maintenance therapy. Sintilimab was administered at a fixed dose of 200 mg, once every 3 weeks, for 2 years until disease progression, no additional clinical benefit or unacceptable toxicity (**Figure 1**).

### Follow-up

The tumor response was assessed according to the RECIST: complete response (CR), partial response (PR), stable disease (SD), and progressive disease (PD). Patient follow-ups were conducted every 6 weeks during combined chemotherapy plus CIK cell treatment and every 8–10 weeks during sintilimab maintenance therapy. After completion of the study, follow-up was conducted every 12 weeks. Hematologic and serum chemical measurement were carried out at each cycle of treatment. Radiographic evaluation of the tumor was performed at each follow-up. Tumor response was assessed using computed tomography scans or magnetic resonance imaging scans at the discretion of the investigator. The primary endpoint of this study was to determine the median OS; the secondary endpoints were to assess the ORR, progression-free survival 1 and 2 (PFS1 and PFS2, respectively), and safety. OS was defined as the duration from the signing of informed consent to the date of death (for any reason) or the last follow-up. PFS1 was defined as the duration from the signing of informed consent to the date of tumor progression, or death (for any reason), or the last follow-up. PFS2 was defined as the duration from the first day of sintilimab treatment to the date of tumor progression, death (for any reason), or the last follow-up. ORR is the proportion of participants with CR and PR. Disease control rate (DCR) is the proportion of participants with CR, PR, and SD. The AEs related to treatment were assessed and graded according to the National Cancer Institute Common Terminology Criteria for Adverse Events Version 4.0 (NCI-CTCAE V4.0).

### Statistical analysis

All statistical analyses were performed using Statistical Product and Service Solutions 22.0 (SPSS22, IBM, NY, USA). Descriptive statistical methods were used to summarize patient characteristics, treatment-related AEs, and treatment response. The Kaplan-Meier method was used to calculate OS, PFS1, and PFS2.

## Results

### Patient characteristics

Between June 2019 and December 2021, 13 patients were enrolled in this phase II study. Among the 13 patients, 10 were men, and three were women, with a median age of 63 years (range: 51–70 years). The baseline characteristics of all patients are listed in **Table 1**. Four patients completed six cycles of chemotherapy, and the remaining patients completed 3–5 cycles of chemotherapy due to adverse reactions to chemotherapy drugs (n=5) or disease progression (n=4). Four patients changed to carboplatin because of severe digestive tract reactions to cisplatin. All patients underwent CIK cell (3–6 cycles) infusion therapy synchronously with chemotherapy, except one patient who did not receive CIK cell infusion for two cycles due to coronavirus disease (COVID-19). After completing chemotherapy combined with CIK cell therapy, nine patients reached stable disease or responded well and were subjected to maintenance treatment with sintilimab.

**Table 1:** Baseline characteristics of patients and the clinical efficacy.

Patient number	Sex/age	Metastasis sites	Cycles efficacy	PFS1(months)	Sintilimab	PFS2(months)	OS(months)
**1**	M/60	Liver, lymph nodes	SD	4.9	Yes	1.3	Died, 9.5
**2**	M/61	Lung, lymph nodes	PR	5.5	Yes	1.7	Alive, 30.1
**3**	F/51	Liver, pleura, lymph nodes	PR	7.9	Yes	3.4	Died, 11.3
**4**	M/68	Brain, lung, lymph nodes	PR	12	Yes	7.3	Alive, 28.3
**5**	M/70	Liver, pleura, bone, lymph nodes	PR	9	Yes	5.9	Died, 22.2
**6**	M/66	Liver, pancreas, lymph nodes	PR	5.2	No		Died, 8.4
**7**	F/63	Liver, lymph nodes	PR	10.2	Yes	4.3	Alive, 24.1
**8**	M/59	Lung, lymph nodes	PR	3.6	No		Died, 20.5
**9**	M/69	Liver, lymph nodes	CR	5.5	Yes	2.3	Died, 11.8
**10**	M/63	Lung, lymph nodes	PR	6.9	Yes	2.1	Died, 11.1
**11**	F/52	Lung, liver, bone, paranephros, lymph nodes	SD	3.5	No		Died, 8.5
**12**	M/62	Lung, liver, lymph nodes	SD	3	No		Died, 5.1
**13**	M/67	Lung, bone	PR	No progress	Yes		Alive, 6.2

### CIK cell characteristics

Harvested CIK cells at each dose contained approximately 5 × 10^9^ cells, of which more than 95% were live cells. It was confirmed that all products were free of microbial contamination. The endotoxin level was less than five endotoxin units per milliliter. Finally, the CIK cells were suspended in 200 mL of saline solution supplemented with 2% human serum albumin and waited for infusion.

### Efficacy

**Figure 2:**
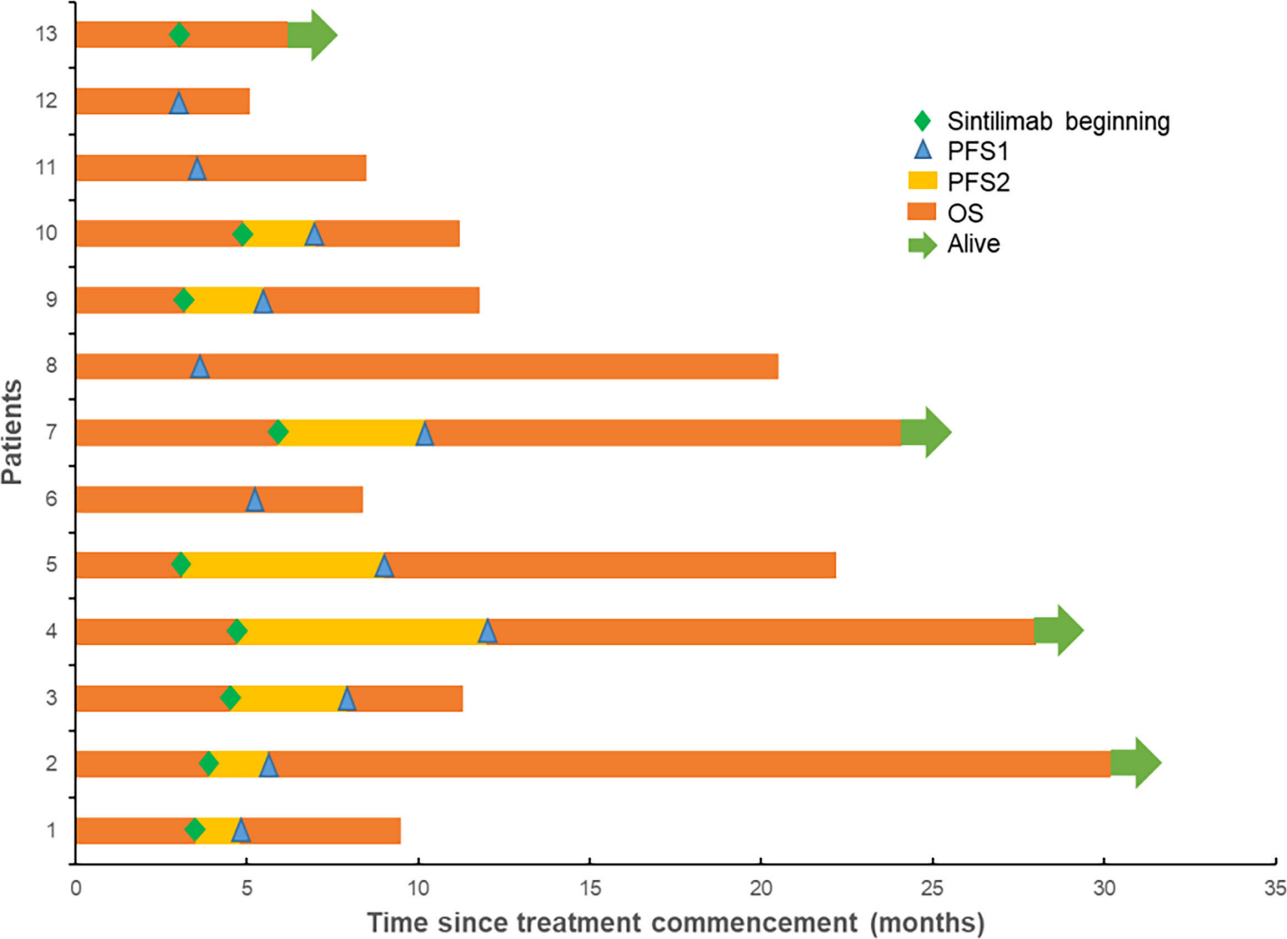
Treatment and survival of ES-SCLC patients.

**Figure 3:**
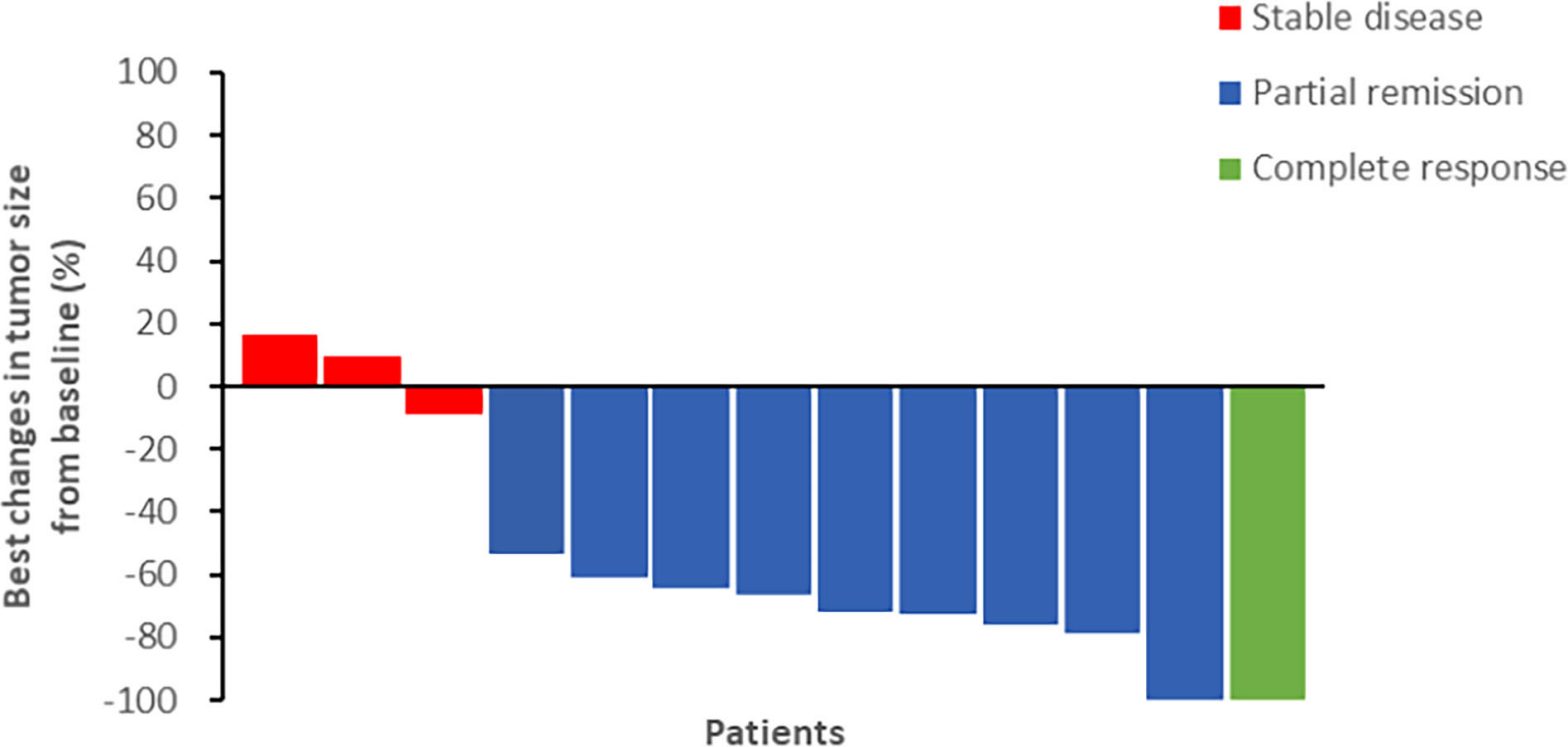
The efficacy of chemotherapy plus CIK cells followed by sintilimab (percentage changes of tumor burden by best response).

**Figure 4:**
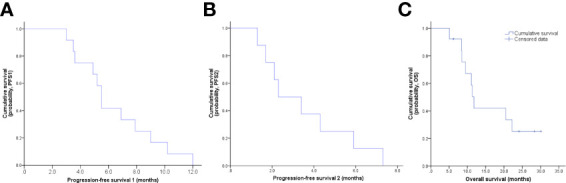
**(A)** PFS1 curve, the median PFS1 was 5.5 months; **(B)** PFS2 curve, the median PFS2 was 2.3 months; **(C)** OS curve, the median OS was 11.8 months.

The final follow-up was on December 14, 2021, and the median follow-up was 24.1 months (range: 6.2–30.5 months). At the data cutoff, nine patients had died, leaving four living participants. Of the nine patients who died, eight died due to disease progression, and one died of an unexplained increase in blood sugar level (**Figure 2**). The median OS was 11.8 (95% confidence interval [CI]: 10.6–13.0) months, median PFS1 was 5.5 (95% CI: 5.0–6.0) months, and the median PFS2 was 2.3 (95% CI: 0.5–4.1) months. The ORR across the 13 enrolled patients was 76.9% (10/13), including 1 (7.7%) with CR and 9 (69.2%) with PR. The DCR was 100%, and the 20-month survival rate was 41.7%. Finally, tumor shrinkage of patients in remission exceeded 50% (**Figure 3**). **Figures 4A–C**, show the PFS1, PFS2, and OS curves, respectively.

### Safety

Grade 3 or 4 AEs related to chemotherapy plus CIK cell (8/13) treatment included anemia and thrombocytopenia; while grade 1 or 2 AEs were mainly nausea, vomiting, and leukopenia. There were only grade 1 AEs thought to be due to CIK cells (3/13), including one case of rash, one case of anorexia, and one of fatigue. Grade 3 AE related to sintilimab occurred in one patient. Therefore, elevation of aminotransferase level and sintilimab infusion were suspended. After treatment with methylprednisolone, the aminotransferase level returned to normal, and sintilimab administration was reinitiated. No adverse reactions recurred after the sintilimab rechallenge. During the follow-up, we learned that one patient died from an unexplained increase in blood glucose (**Table 2**).

**Table 2:** Treatment related toxicity.

Event	CIK cells plus Chemotherapy		Sintilimab	
	Grade 1 or 2	Grade 3 or 4	Grade 1 or 2	Grade 3 or 4
**Anemia**	6	7	0	0
**Thrombocytopenia**	4	2	0	0
**Leukopenia**	12	0	0	0
**Nausea**	13	0	0	0
**Vomiting**	9	0	0	0
**Constipation**	1	0	0	0
**Diarrhea**	2	0	0	0
**Fatigue**	2	0	0	0
**Dizziness**	2	0	0	0
**Hyponatremia**	2	0	0	0
**Creatinine increased**	3	0	0	0
**Hyperuricemia**	3	0	0	0
**Anorexia**	2	0	0	0
**Maculopapule**	1	0	0	0
**Aminotransferase increased**	0	0	0	1

## Discussion

The prognosis of ES-SCLC patients is dismal, with a median OS of patients who have not received anti-tumor treatment of only 2 to 4 months (26). Previously, the standard therapeutic approach for ES-SCLC was chemotherapy, resulting in a median OS of 7–10 months. The emergence of ICIs improved the prognosis of ES-SCLC patients, prolonging the median OS to over one year, for the first time, with the addition of anti-PD-L1 to first-line chemotherapeutic regimens. Despite this, the prognosis of ES-SCLC patients is still very poor and needs improvement, and thus it is very important for researchers to explore novel therapeutic regimens for ES-SCLC patients. In this study, the combination of chemo- and immunotherapy resulted in an ORR of 76.9%, DCR of 100%, a 20-month survival rate of 41.7%, and a median PFS1 of 5.5 months. These results suggest that this regimen may be a safe and effective novel treatment for ES-SCLC patients.

In the first-line treatment of ES-SCLC patients, results were unsatisfactory for anti-PD-1 antibodies and positive for anti-PD-L1 antibodies. In the KEYNOTE-604 study, the median OS was 10.8 months in the pembrolizumab plus etoposide and platinum group versus 9.7 months in the placebo plus etoposide and platinum group. For the two groups, the median PFS was 4.5 months and 4.3 months, respectively, and the ORR was 70.6% and 61.8%, respectively (27). In the IMpower133 trial, the median OS was 12.3 months for the atezolizumab group and 10.3 months for the placebo group. For these two groups, the median PFS was 5.2 months and 4.3 months, respectively, and the ORR was 60.2% and 64.4%, respectively (28). In the CASPIAN study, the median OS was 13.0 months in the durvalumab plus platinum-etoposide group versus 10.3 months in the platinum-etoposide group. For the two groups, the median PFS was 5.1 months and 5.4 months, respectively, and the ORR was 67.9% and 57.6%, respectively (9). After completing the clinical studies described above, the FDA approved atezolizumab or durvalumab plus chemotherapy as the first line of treatment for ES-SCLC. A comparison of our results with those of the IMpower133 and CASPIAN studies reveals that the median OS identified in our research is between that observed in the chemotherapy group and the experimental group, the ORR is slightly better, and the median PFS values are similar.

The high ORR (76.9%) and DCR (100%) values obtained in our study may be due to CIK cells promoting a positive response to chemotherapy in ES-SCLC patients. For example, cytotoxic drugs can cause the release of tumor antigens by attacking tumor cells, thereby exposing more epitopes and upregulating the susceptibility of immune effector cells (29). Furthermore, this interaction can interfere with the tumor microenvironment, trigger remodeling of vascularization, and allow cytotoxic T cells to circulate into tumor cells (30–32). In contrast, CIK cells can reduce bone marrow suppression caused by chemotherapy, restore immune balance, reverse chemoresistance, and even directly kill chemo-surviving cancer stem cells (33–36). Thus, they promote each other in their activity and fight tumor cells together. Liu et al. (16) reported that a randomized, multi-center, open-label trial of CIK cells plus chemotherapy achieved great success in treating squamous non-small cell lung cancer (NSCLC). In this study, the PFS, OS, and ORR of the combined treatment group are better than the chemotherapy group alone. The AEs observed, of any grade, were not different between the two groups. Collectively, the results of this clinical study and our study jointly verify that combining CIK cells with chemotherapy is feasible, and causes mild adverse reactions.

The median PFS after first-line chemotherapy for ES-SCLC is in the range of only 1.4–2.1 months (10, 37). Researchers have tried various methods of maintenance treatment after initial chemotherapy to prolong PFS. Unfortunately, a phase II study involving the use of pembrolizumab for maintenance treatment in ES-SCLC patients, who did not progress under first-line platinum-based chemotherapy, did not produce a desirable result. The median PFS was only 1.4 months (38). Similarly, the CheckMate451 was a global, double-blind, phase III study of nivolumab plus ipilimumab, nivolumab alone, or placebo as maintenance therapy in patients with ES-SCLC, also failed. In this study, the median PFS was 1.7, 1.9, and 1.4 months, respectively (10).

The PFS of maintenance treatment using anti-PD-1 therapy in our study was 2.3 months (95% CI: 0.5–4.1), longer than that of pembrolizumab and nivolumab, and may be related to CIK cells. We speculate that the mechanism of action may include the following factors: First, CIK cells, which are a mixture of T lymphocytes, perform non-MHC-restricted killing of tumors through the differentiated CD3^+^CD56^+^ subset (11). PD-1 blockade therapy mainly kills tumor cells by activating effector T cells and entering the tumor microenvironment. Pre-clinical studies have shown that ICIs can promote the proliferation of CIK cells and increase toxicity (39, 40). In a clinical study, Pan et al. found that CIK cells plus chemotherapy-treated patients with metastatic colorectal cancer with an increased number of CD3^+^CD56^+^ subgroups had a better OS rate than those with a reduced number of CD3^+^CD56^+^ subgroups (41). In addition, Han et al. showed that combination therapy with CIK cells plus a PD-1 blocking antibody might increase CD3^+^ CD16^+^ CD56^+^ T cells, reversing the resistance to anti-PD-1 antibody treatment, and enhancing the clinical response in patients with NSCLC (42). Second, CIK cells may be transformed into more effector T cells under the action of anti-PD-1 therapy; however, it is not clear whether this effect occurs in the periphery or if CIK cells infiltrate the tumor microenvironment. Third, the increase in the total number of CD56^+^ cells may contribute to combination therapy, including CIK cells and anti-PD-1 antibodies (17, 43). These factors may also account for the longer 20-month survival rates (41.7%) in our study.

NSCLC patients treated with PD-1/L1 inhibitors can exhibit considerable long-term survival; this rarely occurs in SCLC. Therefore, it is particularly important to screen the dominant population for immunotherapy. In our study, four patients were tested for PD-L1, using the standard 22C3 antibody (Roche), and all were negative. However, one of the four patients had no disease progression. A meta-analysis showed that a high neutrophil-to-lymphocyte ratio (NLR) before treatment is negatively correlated with SCLC prognosis (44). The only patient with an enlarged tumor in our study had a pre-treatment NLR of 12.7, while the highest NLR in the above meta-analysis was 5.

This study has some limitations. First, the number of patients enrolled is small, with slow enrollment speed and poor representativeness. This may be due to the relatively low incidence of ES-SCLC and many clinical trials being carried out simultaneously. Moreover, due to the impact of COVID-19, only six patients were enrolled in 2020–2022. Second, the patients in our study were generally in poor condition when investigators initiated this clinical study. For example, our study included patients with symptomatic brain metastasis, even though patients with symptomatic brain metastasis are generally excluded from clinical studies. Third, due to the impact of COVID-19, some patients failed to return to the hospital on time, resulting in treatment delays, leading to disease progression in some patients due to long treatment intervals. This may have underestimated treatment efficacy. Increased patient participation and longer follow-up times are needed to confirm the efficacy and safety of anti-PD-1 antibody maintenance treatment of ES-SCLC after first-line chemotherapy combined with CIK cells. Despite this, the high ORR and DCR values and longer OS obtained in this study provide new treatment options for the treatment of ES-SCLC.

## Conclusions

This prospective study shows that chemotherapy combined with CIK cell therapy results in higher ORR and DCR. Compared with historical data, the 20-month survival rates were higher, and the median PFS2 was improved with sintilimab maintenance therapy. Although the median OS in our study was 11.8 months, the PFS1 seems comparable to that observed in the IMpower133 and CASPIAN regimens. In addition, this treatment regimen was tolerated well, and the results of our study provide additional therapeutic approaches for ES-SCLC patients.

## Data availability statement

The original contributions presented in the study are included in the article/supplementary material. Further inquiries can be directed to the corresponding authors.

## Ethics statement

The studies involving human participants were reviewed and approved by Ethics Committee of Henan Cancer Hospital. The patients/participants provided their written informed consent to participate in this study.

## Author contributions

QG and LZ designed the study. BM, YuZ, YoZ, YS, XF, BX, JG, YY, FZ, MZ, HH, FL, HL, and ZW collected the data. BM and LZ analyzed and interpreted the data. BM drafted the article, which was revised by QG and LZ. QG approved the version to be published. All authors read and provided comments on the article. All authors contributed to the article and approved the submitted version.

## Funding

The work was supported by the Henan Province Industry-University-Research Cooperation Project (Grant No. 182107000027).

## Acknowledgments

The authors thank Editage (www.editage.cn) for English language editing.

## Conflict of interest

The authors declare that the research was conducted in the absence of any commercial or financial relationships that could be construed as a potential conflict of interest.

## Publisher’s note

All claims expressed in this article are solely those of the authors and do not necessarily represent those of their affiliated organizations, or those of the publisher, the editors and the reviewers. Any product that may be evaluated in this article, or claim that may be made by its manufacturer, is not guaranteed or endorsed by the publisher.

## Abbreviations

AE, adverse event; CI, confidence interval; CIK, cytokine-induced killer; CR, complete response; DCR, disease control rate; ES-SCLC, extensive-stage small-cell lung cancer; NSCLC, non-small cell lung cancer; ORR, objective response rate; PD-1, programmed death 1; PFS, progression-free survival; PR, partial response.
